# A strategy to ensure safety of stem cell-derived retinal pigment epithelium cells

**DOI:** 10.1186/s13287-016-0380-6

**Published:** 2016-09-02

**Authors:** Parul Choudhary, Paul John Whiting

**Affiliations:** 1 Pfizer Neuroscience and Pain Research Unit, The Portway, Granta Park, Great Abington, Cambridge, CB21 6GS UK; 2 Present Address: AR-UK Drug Discovery Institute, University College London, London, WC1E 6BT UK

**Keywords:** Retinal pigment epithelium, Embryonic stem cells, Flow sorting, Age-related macular degeneration, Cell surface markers

## Abstract

Cell replacement and regenerative therapy using embryonic stem cell-derived material holds promise for the treatment of several pathologies. However, the safety of this approach is of prime importance given the teratogenic potential of residual stem cells, if present in the differentiated cell product. Using the example of embryonic stem cell-derived retinal pigment epithelium (RPE) for the treatment of age-related macular degeneration, we present a novel strategy for ensuring the absence of stem cells in the RPE population. Based on an unbiased screening approach, we identify and validate the expression of CD59, a cell surface marker expressed on RPE but absent on stem cells. We further demonstrate that flow sorting on the basis of CD59 expression can effectively purify RPE and deplete stem cells, resulting in a population free from stem cell impurity. This purification helps to ensure removal of stem cells and hence increases the safety of cells that may be used for clinical transplantation. This strategy can potentially be applied to other pluripotent stem cell-derived material and help mitigate concerns of using such cells for therapy.

**Electronic supplementary material:**

The online version of this article (doi:10.1186/s13287-016-0380-6) contains supplementary material, which is available to authorized users.

## Introduction

Safety is a primary consideration for any clinical programme. Differentiated cell products generated from human embryonic stem cells (hESCs) or human induced pluripotent stem cells (hiPSCs) pose particular challenges because there is a risk that residual pluripotent stem cells could give rise to teratomas upon transplantation [1, 2]. However, the therapeutic promise of using stem cell-derived material cannot be overlooked. For instance, the transplantation of pluripotent-derived retinal pigment epithelium (RPE) in patients suffering from age-related macular degeneration (AMD) has the potential to halt visual decline and restore visual function. This approach has been under investigation in both pre-clinical and clinical settings with promising results [3, 4].

AMD is a leading cause of irreversible vision loss among the older population in the developed world [5]. AMD affects RPE cells, which are situated as a monolayer beneath the photoreceptors and perform several important functions to maintain the visual cycle; for example, metabolism and storage of retinoid, phagocytosis of rod outer segments, absorption of scattered light, barrier activity and ion transport [6]. Degeneration of RPE in AMD results in subsequent loss of photoreceptors leading to loss of vision. Several methods for generating mature and functional RPE from hESC and hiPSC have been described in the literature (reviewed in [7]). Techniques such as quantitative PCR, flow cytometry and in-vivo teratoma formation are commonly employed to show that the RPE population is free from hESC or hiPSC [8]. In this report, we investigate a strategy that enables us to select RPE cells and purify them from any potential stem cell contaminant, thereby ensuring safety for clinical application. Using an unbiased screening approach we identify CD59, a cell surface marker that is expressed on RPE but is absent on hESC. CD59, also known as protectin, is an 18–21 kDa glycoprotein which anchors to cell membranes by a GPI anchor [9]. It inhibits the membrane attack complex (MAC) which mediates cell lysis and inflammation and is formed upon complement activation [10]. Interestingly, MAC formation correlates with AMD severity and its negative regulation by CD59 is under investigation for AMD therapy [11, 12]. CD59 has been shown previously to be expressed in RPE cells in both human and rodent models of retinal physiology [13–18].

We show that sorting cells on the basis of CD59 expression positively selects RPE cells and concomitantly removes pluripotent stem cells from a mixed population, thereby resulting in purification. This approach can potentially be applied to diverse cell types and can help to ensure safety of stem cell-derived cells in general or be used to generate homogeneous cell populations for research and in-vitro disease modelling.

## Materials and methods

### Directed differentiation of pluripotent stem cells towards RPE

The protocol for differentiation of hESC or hiPSC towards RPE has been described elsewhere [19]. Briefly, pluripotent stem cells are seeded on Matrigel (Corning) coated surfaces and on day 2 of culture the growth medium (KnockOut DMEM medium (Gibco) supplemented with 20 % KnockOut Serum Replacement Xeno-Free (Gibco), 1 % β-mercaptoethanol (Sigma), 1 % GlutaMax (Gibco) and 1 % non-essential amino acid solution (Gibco)) is supplemented with two inhibitors, 1 μM LDN-193189 (Stemgent) and 10 μM SB-431542 (Sigma), for a period of 4 days. This is followed by treatment of cells with 100 ng/ml BMP 4/7 (R&D Systems) for a period of 3 days. At this stage, cells are dissociated and replated in the presence of 100 ng/ml Activin A (R&D Systems). After a period of 19 days, cells are again dissociated and replated in medium without any supplements for 14 days. By this stage of the protocol, RPE cells with close to 100 % purity are generated. Interested readers can refer to a patent (US 2015/0159134 A1) that also describes the protocol in greater detail. In this report, we used the pluripotent stem cell line SHEF1 for differentiation towards RPE.

### Imaging and flow cytometry

RPE cells derived by directed differentiation were plated at a density of 100,000 cells/cm^2^ on 384-well plates coated with Matrigel (BD Biosciences). Cells were cultured for 7 days before performing a screen for cell surface protein expression using the BD Lyoplate™ Human Cell Surface Marker Screening Panel (BD Biosciences). The morphology of cells used for secreening is shown in Additional file 1: Figure S1. The manufacturer’s recommendations for bioimaging were followed for screening cells. For flow cytometry, cells were stained with the Live/Dead fixable green dead cell stain kit (Invitrogen). Following this, cells were fixed with 1 % PFA and washed three times with PBS. Centrifugation was performed at 300 × *g* for 5 minutes. Cells were resuspended to approximately 1 × 10^6^ cells/100 μl in PBS containing 2 % BSA. Cells were stained with PE-conjugated or APC-conjugated antibodies (BD Pharmingen) using 20 μl antibody per 100 μl of experimental sample. Samples were incubated for 30 minutes protected from light at room temperature, and then washed twice before being resuspended in 150 μl PBS containing 2 % BSA for analysis on the Accuri C6 flow cytometer. Negative controls consisting of unstained cells and cells stained with the isotype control (BD Pharmingen) were performed in parallel. Flow cytometry analysis was performed by gating out the debris and doublets and selecting live. Sorting was performed under sterile conditions using an inFlux v7 cytometer housed in a biological safety cabinet. The sorting efficiency (i.e. number of positive events detected by the cytometer compared with the number of events around which a sort decision was made) was between 80 and 85 %.

### RNA extraction, cDNA synthesis and quantitative PCR

Total RNA was extracted from RPE cells using the RNeasy Mini or Micro Kit (Qiagen) with on-column DNase digestion. cDNA was synthesized using the High Capacity cDNA Synthesis kit (Applied Biosystems). Individual gene expression was assessed using predesigned Taqman assays (Applied Biosystems) and the reactions were carried out on the CFX96 iCycler platform (Biorad). Gene expression in all instances was quantified by the relative quantification method of 2^–ΔΔCt^ and normalized to geometric means of at least two housekeeping genes.

## Results

### Screening to identify cell surface markers expressed on RPE cells

**Fig. 1:**
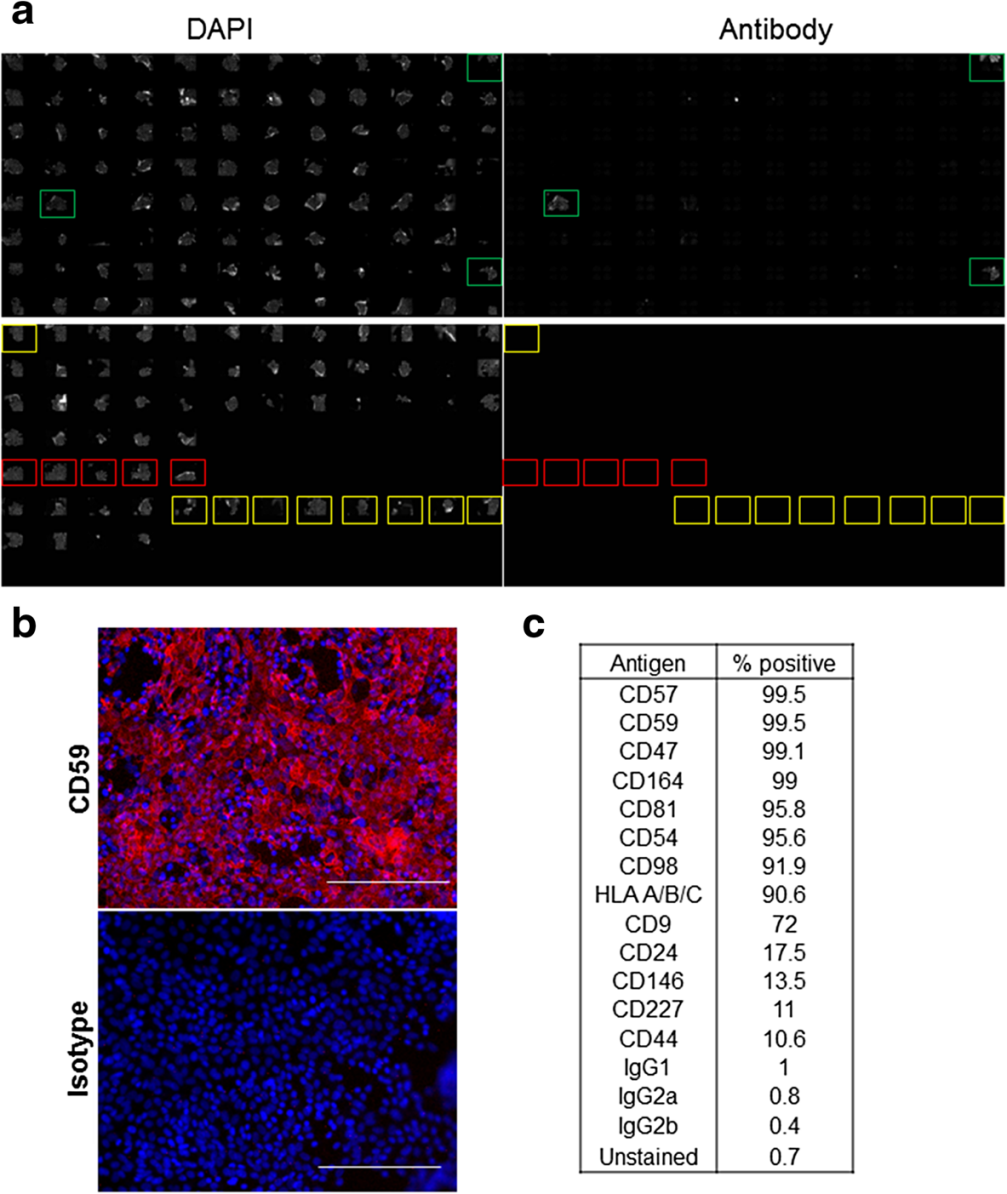
Screening for cell surface markers expressed on RPE cells. **a** Representative image showing results of screening for identification of cell surface markers expressed on RPE. Overview of DAPI (*left*) and antibody (*right*) specific staining in a 384-well plate. *Green boxes* indicate positive staining with a cell surface marker, *red boxes* indicate isotype controls and *yellow boxes* indicate unstained cells. **b** Representative image showing a magnified view of a well staining positive with an antibody against CD59 (*red*) compared with background staining using isotype control. Nuclei are stained with DAPI (*blue*). *Scale bar* = 150 μm. **c** % positive expression of indicated antigen in RPE cells as determined by flow cytometry. *DAPI* 4′,6-diamidino-2-phenylindole (Colour figure online)

To identify a unique cell surface marker expressed on RPE cells, we performed an unbiased screen for cell surface markers that were present exclusively on mature RPE but not on hESC or progenitor cells to enable effective depletion of these impurities by cell sorting. For this approach, we made use of the BD Lyoplate™ Human Cell Surface Marker Screening Panel consisting of a library of antibodies targeting a range of cell surface proteins, glycoproteins and glycosphingolipids together with relevant isotype controls. Immunocytochemistry was performed in live cells, to prevent fixation-induced artefacts, and under non-permeabilized conditions so that only proteins expressed on the cell surface could be visualized. Using this approach, we found 13 ‘hits’ or markers staining positively on RPE cells above background levels using negative controls, for example isotype matched antibodies and unstained cells (Fig 1a). An example of immunostaining of a positive hit, CD59, is shown in Fig. 1b. Next, we used flow cytometry to verify expression of markers identified by immunocytochemistry because it can be more easily adapted to cell sorting and purification applications. Of the 13 markers tested, four markers were found to be expressed at low levels (<20 %) whereas the remaining nine markers had >90 % positive expression compared with a range of isotype controls (Fig. 1c). We excluded markers that are known to be ubiquitously expressed on all nucleated cells (e.g. HLA) or on tumour cells (e.g. CD47) and focused our attention on five markers (CD57, CD59, CD81, CD164 and CD98) for further interrogation.

### CD59 is expressed on RPE and not on hESC 

**Fig. 2:**
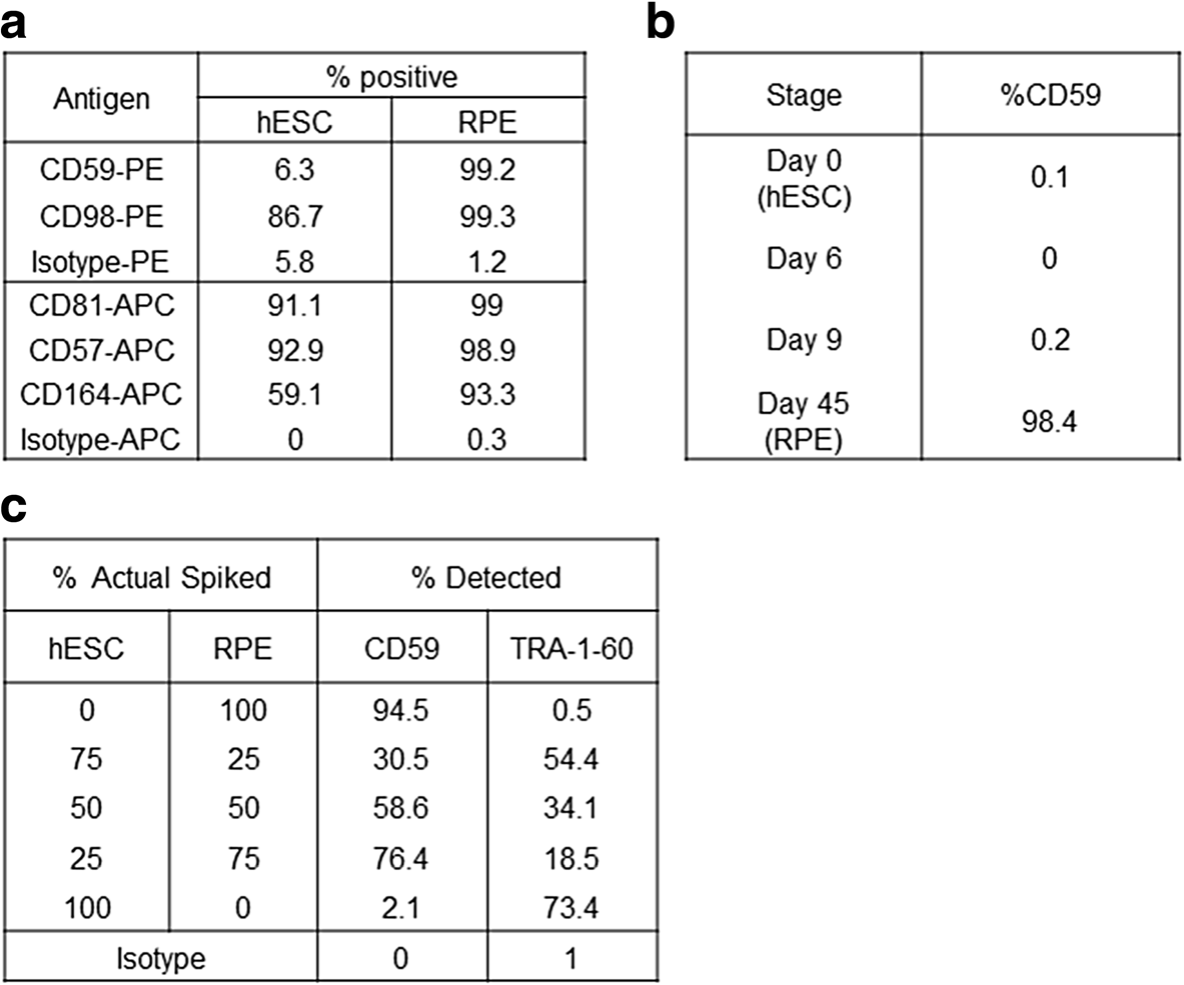
Identification and validation of CD59 expression on RPE cells. **a** % positive expression of indicated antigens in hESCs and RPE cells as determined by flow cytometry. PE-conjugated or APC-conjugated antibodies together with the respective isotypes were used. **b** % positive CD59 expression at different stages of the RPE differentiation protocol. **c** Results of a spiking experiment where different ratios of hESCs and RPE were mixed together and expression of CD59 and the stem cell marker TRA-1-60 was determined by flow cytometry. *hESC* human embryonic stem cell, *RPE* retinal pigment epithelium

For application of cell sorting to purify RPE away from any residual hESC, the cell surface marker of choice should be expressed on RPE but not on stem cells. Therefore, we next tested whether the shortlisted markers fulfilled this criterion. The hESC line SHEF1 together with RPE derived from it were tested in parallel for expression of markers of interest by flow cytometry. Out of the markers tested, only CD59 was found to be expressed on RPE but at very low levels on hESC when compared with the corresponding isotype control (Fig. 2a, Additional file 2: Figure S2). Furthermore, negligible expression of CD59 was detected during initial stages of differentiation where the hESC are not yet committed to an RPE fate (Fig 2b, Additional file 3: Figure S3). This supports the notion that sorting based on CD59 expression could purify RPE away from stem or progenitor cells.

In order to further explore specificity of CD59 expression on RPE compared with hESC, we performed a spiking experiment where a cell suspension consisting of known numbers of hESCs mixed with known numbers of RPE cells was created. Flow cytometry was used to detect CD59 expression in this mixed cell population and TRA-1-60 expression was used to identify stem cells. We found the CD59-positive staining to track closely with the proportion of RPE cells present in the cell suspension (Fig 2c, Additional file 4: Figure S4). This confirms that CD59 expression can be used to distinguish between RPE and hESC and can potentially be used to remove stem cells from RPE cultures.

### Negative selection for CD59 as a strategy for removal of stem cells and purification of viable RPE

**Fig. 3:**
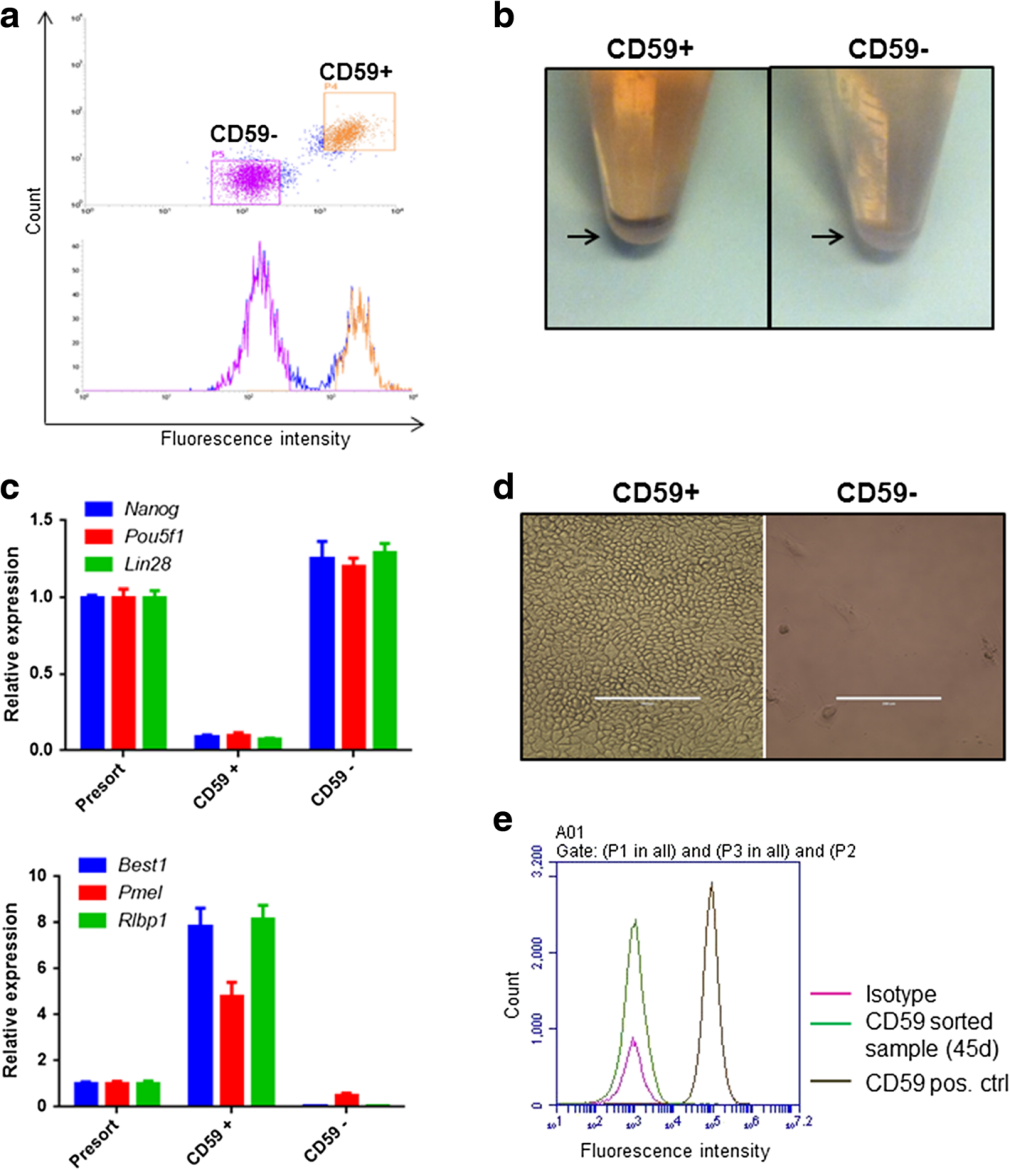
Sorting for CD59 for RPE purification and stem cell removal. **a** Use of flow cytometry-based sorting to collect the population expressing CD59 (*CD59+*) separately from the population not expressing CD59 (*CD59–*) in a 1:1 mixture of hESCs and RPE cells. **b** Representative image showing pigmentation in the cell pellets obtained from the CD59+ and CD59– fractions. **c** Quantitative PCR to measure expression of pluripotency (*top*) and RPE markers (*bottom*) in CD59+ and CD59– fractions. The pre-sorted cell suspension is used for comparison. *ACT* and *GAPDH* were used as housekeeping genes (*n* = 4, ± SD). **d** Representative bright-field images showing the cobblestone architecture of CD59+ and CD59– fractions seeded at a density of 78,000 cells/cm^2^ and cultured for a period of 45 days post sorting. *Scale bar* = 200 μm. **e** No CD59-PE fluorescence can be seen in cells described in **d** as compared with cells freshly stained for CD59 used as a positive control (*CD59 pos. ctrl*)

In order to demonstrate a proof of principle supporting sorting on the basis of CD59 expression to deplete stem cells and purify RPE, we created a cell population consisting of hESCs and RPE mixed in a 1:1 ratio. Flow cytometry was used to collect the population staining positive for CD59 separately from the population staining negative for CD59 expression (Fig 3a, Additional file 5: Figure S5). Encouragingly, the cell pellets of the CD59-positive fraction displayed pigmentation consistent with the presence of RPE whereas the CD59-negative cells were non-pigmented, indicative of hESCs (Fig 3b). Quantitative PCR was used to analyse the expression of stem and RPE markers in the two fractions. Consistent with the visual pigmentation, hESC markers (*Pou5f1*, *Nanog*, *Lin28*) were found to be expressed in the CD59-negative fraction whereas RPE markers (*Best*, *Rlbp1*, *Pmel*) were present in the CD59-positive fraction (Fig 3c). The sorted CD59-positive cells could be maintained in culture for an extended period and cells adopted a typical RPE morphology (Fig 3d, left). In contrast, the CD59-negative fraction seeded at the same density does not develop into a cellular monolayer under the same culture conditions (Fig 3d, right). Furthermore, the sorted cells did not retain the anti-CD59 antibody used for initial sorting because there was no PE signal detectable in the cells after 45 days in culture (Fig 3e). This suggests that RPE purified through this approach would have a normal cell surface profile with no exogenous antibody present which might interfere with function.

**Fig. 4:**
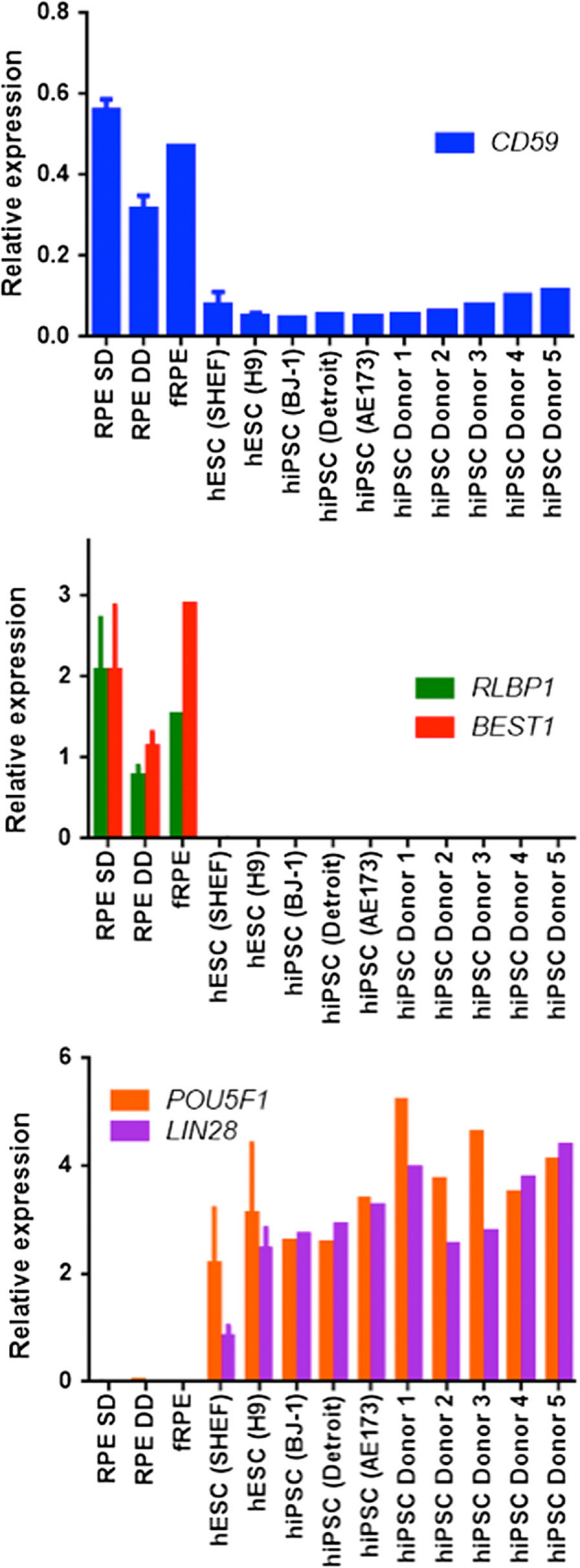
Comparison of CD59 expression in RPE vs pluripotent stem cells. Quantitative PCR-based measurement of expression of *Cd59*, *Best1*, *Rlbp1*, *Pou5f1* and *Lin28* transcript in a variety of cells. *ACTB* and *HPRT* were used as housekeeping genes. *Bars* represent ± SD (*n* = 1–4). *RPE SD* RPE derived by spontaneous differentiation, *RPE DD* RPE derived by directed differentiation, *fRPE* foetal RPE, *hESC* human embryonic stem cell, *hiPSC* human induced pluripotent stem cell, *RPE* retinal pigment epithelium

We further performed quantitative PCR analysis to check expression of CD59 transcript in RPE derived from different sources; for example, the directed differentiation approach used in this study, spontaneous differentiation described previously [20] or in foetal RPE. This RPE CD59 expression was compared with expression in a variety of human pluripotent cell lines ranging from hESC lines (e.g. SHEF1, H9) and hiPSC lines from a variety of donors. RPE markers *Rlbp1* and *Best1* and stem cell markers *Pou5f1* and *Lin28* were used to distinguish between the identity of RPE and stem cells. On average, the expression of CD59 was about 6-fold higher in RPE cells compared with pluripotent cells (Fig 4), indicating that sorting for CD59 could be broadly applied for purification of RPE cells and removal of stem cell impurity irrespective of the type of pluripotent stem cell line used for RPE derivation. Taken together, these data are in keeping with the hypothesis that such sorting approaches can be undertaken to effectively purify stem cells during a differentiation paradigm.

## Discussion

Recent breakthroughs in stem cell biology, especially the development and application of induced pluripotent stem cell techniques, have generated tremendous enthusiasm and efforts to explore the therapeutic potential of stem cells in regenerative medicine. The use of stem cell-derived RPE for the treatment of AMD is under clinical investigation because there are several advantages of targeting the eye as an organ for stem cell-based therapies, for example ease of administration route, size, potential immune privilege, separation from systemic circulation and so forth [21]. However, in applications based on hESC or hiPSC, the safety of the therapeutic product is of prime importance given that residual stem cells may have the capacity of unlimited proliferation and self-renewal resulting in teratomas or teratocarcinomas that can potentially be highly malignant [2].

Current protocols for RPE generation from hESC or hiPSC rely on the differentiation process together with culture conditions; for example, use of culture medium and extracellular matrices that would not support stem cell growth to prevent the presence of residual stem cells in a differentiated RPE population. Furthermore, most cell purifications performed for clinical cell therapy utilize antibody-coupled magnetic bead-based sorting, referred to as magnetic-activated cell sorting (MACS) [22, 23], a population-based method which has a faster purification time compared with FACS. However, the method is limited by a lower efficiency of sorting and does not allow for analysis of individual cells. This is a disadvantage particularly for hESC or hiPSC applications because the benefit of single cell analysis allows a better chance for identifying and isolating stem cells that may be present in a minority.

In this report, we aimed to explore a novel strategy for ensuring purity of a RPE population by identifying and sorting on the basis of a specific cell surface marker that would be expressed on RPE but not on stem cells. We used an unbiased screening approach to identify CD59 expression on RPE but at negligible levels on stem cells and further demonstrated that sorting for CD59 expression can effectively purify RPE and deplete pluripotent stem cells from a mixed population. CD59 is involved in suppression of the complement pathway and contributes towards the potential RPE-dependent immune privilege associated with the eye [18]. CD59 identification is therefore consistent with the functional attributes of RPE. We have not formally ruled out that CD59 is expressed exclusively on RPE and not on other cell types generated during differentiation. However, because sorting for CD59 will lead to removal of stem cells, it is still a beneficial step to be included in the differentiation protocol to ensure that residual stem cells are removed effectively. Further work is also needed to demonstrate that CD59-positive cells do not have any features of pluripotency, for example with the use of teratoma and colony formation assays, embryoid body formation and alkaline phosphatase staining. It will also be interesting to explore strategies based on negative sorting for RPE purification. For instance, pluripotent stem cell specific markers such as SSEA3 or TRA-1-60 could be used to label residual stem cells and the negative fraction would potentially be free of pluripotent stem cells. However, there are technical challenges around the detection of such potentially rare events which require a high signal-to-noise ratio and a large sample number to be accurate.

It is noteworthy that the CD59 transcript can be detected in pluripotent stem cells, albeit at a level that is about 6-fold level lower than that in RPE cells. However, the expression of CD59 protein is negligible in stem cells as demonstrated by our flow cytometry data. This highlights the importance of corroborating transcript levels with protein expression because there may not necessarily be a proportional relationship. In this context, our screen using live, non-permeabilized cells allowed identification of cell surface expressed proteins that were amenable to cell sorting and separation-based applications. Further work is also needed to understand the dynamics of CD59 protein expression during the differentiation time course. This will help to clarify whether progenitors at intermediate stages of differentiation can be separated from mature RPE, in addition to undifferentiated stem cells.

## Conclusion

In summary, this study demonstrates the utility of a novel sorting approach based on CD59 expression that may help to ensure safety of pluripotent stem cell-derived RPE for clinical applications as well as in generation of pure RPE populations for research and in-vitro disease modelling. This approach can also have utility for other stem cell-derived cell types and their therapeutic use.

## Abbreviations

AMD, age-related macular degeneration; CD59, cluster of differentiation 59; hESC, human embryonic stem cell; hiPSC, human induced pluripotent stem cell; MAC, membrane attack complex; RPE, retinal pigment epithelium

## Additional files

Additional file 1: Figure S1. Showing a representative bright-field image of typical morphology of cells used for screening for cell surface markers. The cells form a monolayer and display cobblestone morphology typical of RPE cells. *Scale bar* = 400 μm (10×) and 200 μm (20×). (TIF 8794 kb) Additional file 2: Figure S2. Showing expression of indicated proteins measured by flow cytometry in hESCs (*left*) and hESC-derived RPE (*right*). *x axis* represents the log-fluorescence intensity, *y axis* represents relative cell counts. (TIF 1923 kb) Additional file 3: Figure S3. Showing expression of CD59 measured by flow cytometry at days 0, 6, 9 and 45 of the differentiation time course. *x axis* represents the log-fluorescence intensity, *y axis* represents relative cell counts. (TIF 1704 kb) Additional file 4: Figure S4. Showing expression of CD59 (*top*) and TRA-1-60 (*bottom*) measured by flow cytometry in cell suspensions created by mixing together different hESC-derived RPE and hESCs. *x axis* represents the log-fluorescence intensity, *y axis* represents relative cell counts. (TIF 2661 kb) Additional file 5: Figure S5. Showing the CD59+ and CD59– samples sorted in Fig. 3 re-analysed by flow cytometry to show the purity of the individual fractions collected. The P4 gated events represent the CD59-positive fraction and the P5 gated events represent the CD59-negative fraction. (TIF 2445 kb)

## References

[1] Heslop JA, Hammond TG, Santeramo I (2015). Concise review: workshop review: understanding and assessing the risks of stem cell-based therapies. Stem Cells Transl Med..

[2] Goldring CE, Duffy PA, Benvenisty N (2011). Assessing the safety of stem cell therapeutics. Cell Stem Cell..

[3] Schwartz SD, Hubschman JP, Heilwell G (2012). Embryonic stem cell trials for macular degeneration: a preliminary report. Lancet..

[4] Schwartz SD, Regillo CD, Lam BL (2015). Human embryonic stem cell-derived retinal pigment epithelium in patients with age-related macular degeneration and Stargardt's macular dystrophy: follow-up of two open-label phase 1/2 studies. Lancet..

[5] Friedman DS, O'Colmain BJ, Munoz B (2004). Prevalence of age-related macular degeneration in the United States. Arch Ophthalmol..

[6] Strauss O (2005). The retinal pigment epithelium in visual function. Physiol Rev..

[7] Leach LL, Clegg DO (2015). Concise Review: Making stem cells retinal: methods for deriving retinal pigment epithelium and implications for patients with ocular disease. Stem Cells..

[8] Kuroda T, Yasuda S, Kusakawa S (2012). Highly sensitive in vitro methods for detection of residual undifferentiated cells in retinal pigment epithelial cells derived from human iPS cells. PLoS One..

[9] Davies A, Simmons DL, Hale G (1989). CD59, an LY-6-like protein expressed in human lymphoid cells, regulates the action of the complement membrane attack complex on homologous cells. J Exp Med..

[10] Davies A, Lachmann PJ (1993). Membrane defence against complement lysis: the structure and biological properties of CD59. Immunol Res..

[11] Hageman GS, Luthert PJ, Victor Chong NH (2001). An integrated hypothesis that considers drusen as biomarkers of immune-mediated processes at the RPE-Bruch's membrane interface in aging and age-related macular degeneration. Prog Retin Eye Res..

[12] Cashman SM, Ramo K, Kumar-Singh R (2011). A non membrane-targeted human soluble CD59 attenuates choroidal neovascularization in a model of age related macular degeneration. PLoS One..

[13] Herrmann P, Cowing JA, Cristante E (2015). Cd59a deficiency in mice leads to preferential innate immune activation in the retinal pigment epithelium-choroid with age. Neurobiol Aging..

[14] Luo C, Zhao J, Madden A (2013). Complement expression in retinal pigment epithelial cells is modulated by activated macrophages. Exp Eye Res..

[15] Juel HB, Kaestel C, Folkersen L (2011). Retinal pigment epithelial cells upregulate expression of complement factors after co-culture with activated T cells. Exp Eye Res..

[16] Yang P, Tyrrell J, Han I (2009). Expression and modulation of RPE cell membrane complement regulatory proteins. Invest Ophthalmol Vis Sci..

[17] Kanuga N, Winton HL, Beauchéne L (2002). Characterization of genetically modified human retinal pigment epithelial cells developed for in vitro and transplantation studies. Invest Ophthalmol Vis Sci..

[18] Liversidge J, Dawson R, Hoey S (1996). CD59 and CD48 expressed by rat retinal pigment epithelial cells are major ligands for the CD2-mediated alternative pathway of T cell activation. J Immunol..

[19] Choudhary P, Booth H, Gutteridge A, Surmacz, B, Louca I, Steer J, Kerby J, Whiting PJ (2016). Directing differentiation of pluripotent stem cells towards retinal pigment epithelium lineage. Stem Cells Trans Med in press. 10.5966/sctm.2016-0088 PMC5442825 28191760

[20] Vugler A, Carr AJ, Lawrence J (2008). Elucidating the phenomenon of HESC-derived RPE: anatomy of cell genesis, expansion and retinal transplantation. Exp Neurol..

[21] Whiting P, Kerby J, Coffey P (2015). Progressing a human embryonic stem cell-based regenerative medicine therapy towards the clinic. Philos Trans R Soc Lond B Biol Sci..

[22] Lang P, Schumm M, Taylor G (1999). Clinical scale isolation of highly purified peripheral CD34+ progenitors for autologous and allogeneic transplantation in children. Bone Marrow Transplant..

[23] Wang X, Rivière I (2016). Clinical manufacturing of CAR T cells: foundation of a promising therapy. Mol Ther Oncolytics..

