# Supplementary figures and images for: A strategy to ensure safety of stem cell-derived retinal pigment epithelium cells
Source: Stem Cell Res Ther. 2016 Sep 2;7(1):127. doi: 10.1186/s13287-016-0380-6 (PMC5010679; doi:10.1186/s13287-016-0380-6)

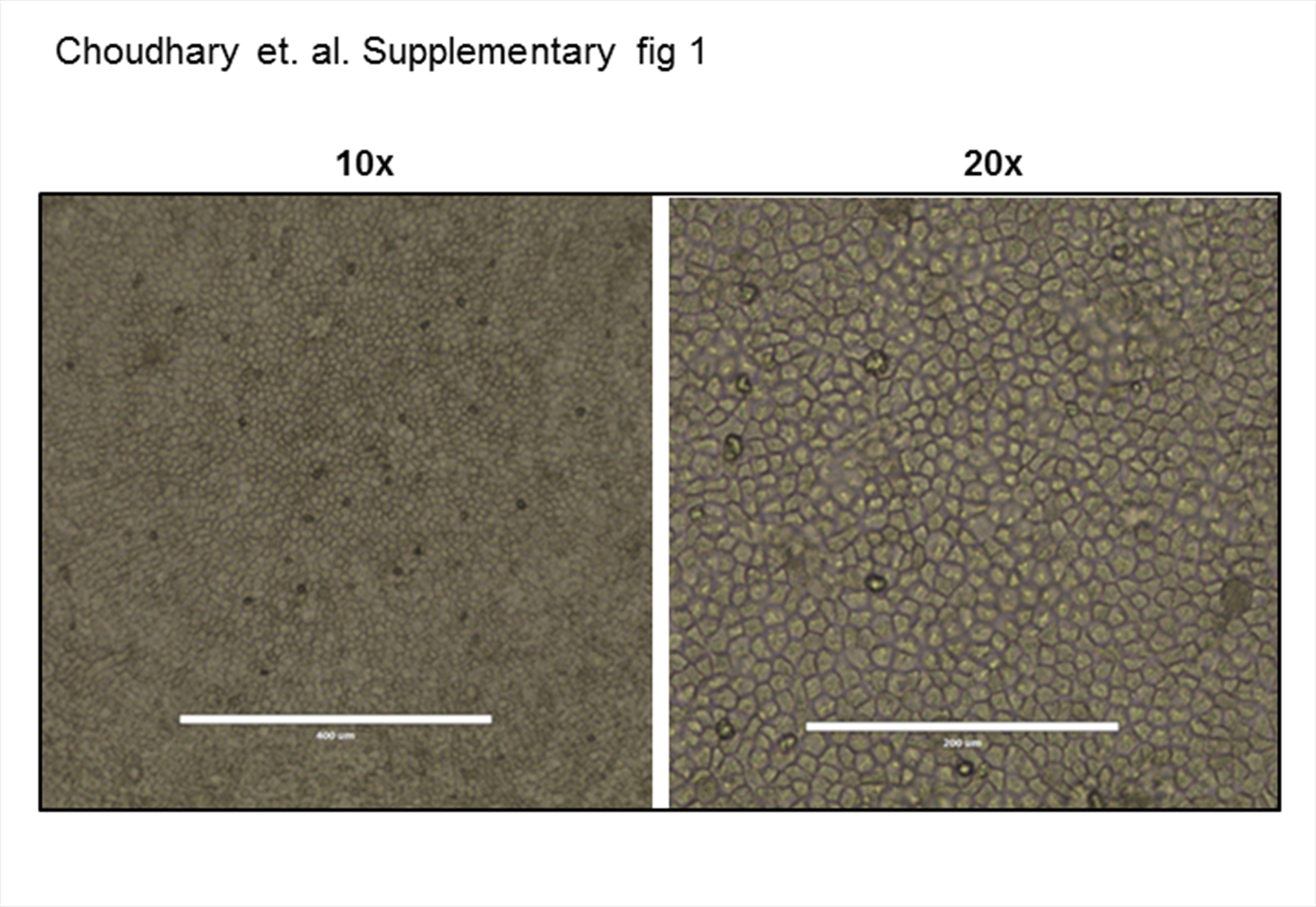

Supplement: Additional file 1: Figure S1. — Showing a representative bright-field image of typical morphology of cells used for screening for cell surface markers. The cells form a monolayer and display cobblestone morphology typical of RPE cells. Scale bar = 400 μm (10×) and 200 μm (20×). (TIF 8794 kb) [file 13287_2016_380_MOESM1_ESM.tif]

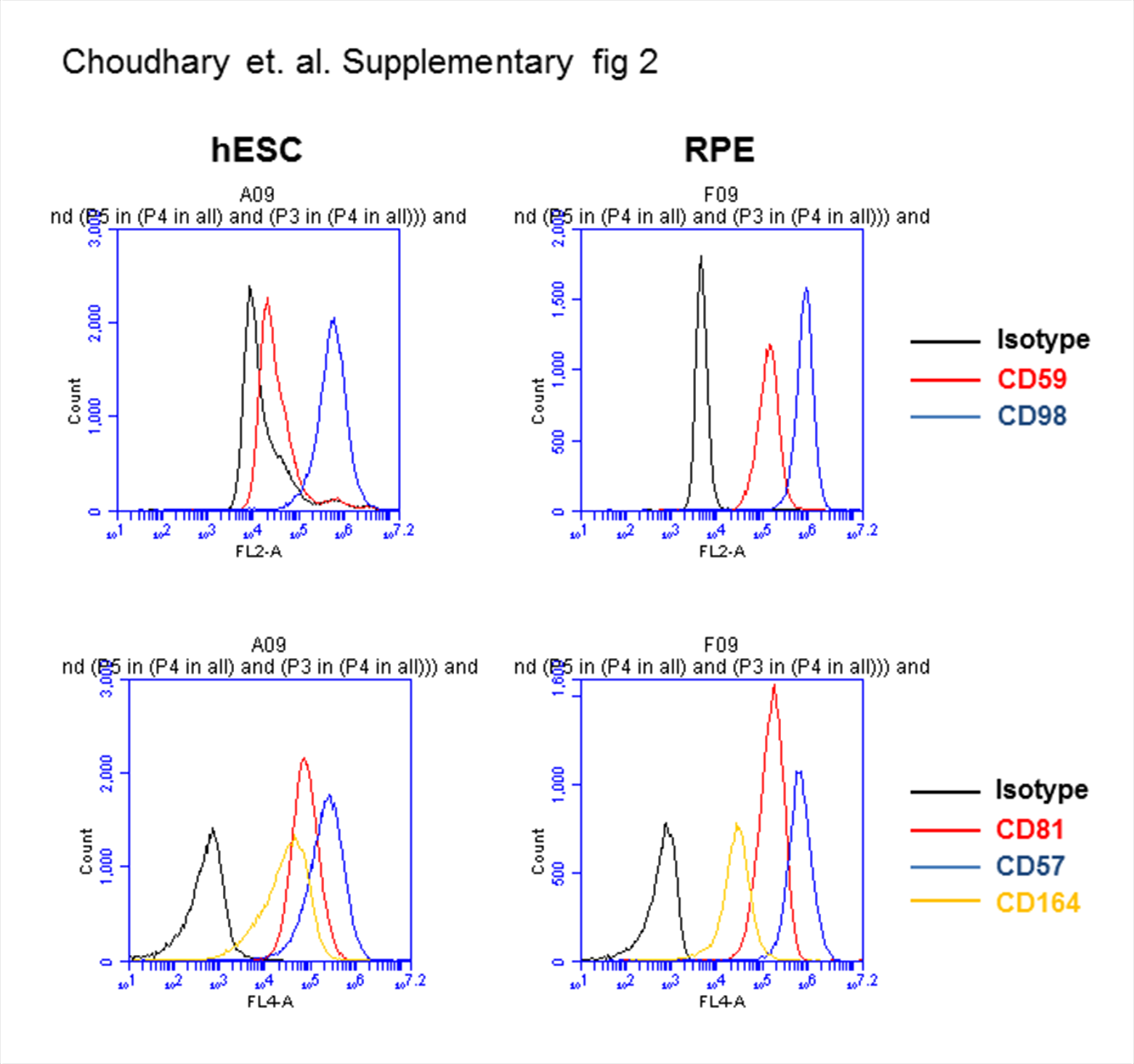

Supplement: Additional file 2: Figure S2. — Showing expression of indicated proteins measured by flow cytometry in hESCs (left) and hESC-derived RPE (right). x axis represents the log-fluorescence intensity, y axis represents relative cell counts. (TIF 1923 kb) [file 13287_2016_380_MOESM2_ESM.tif]

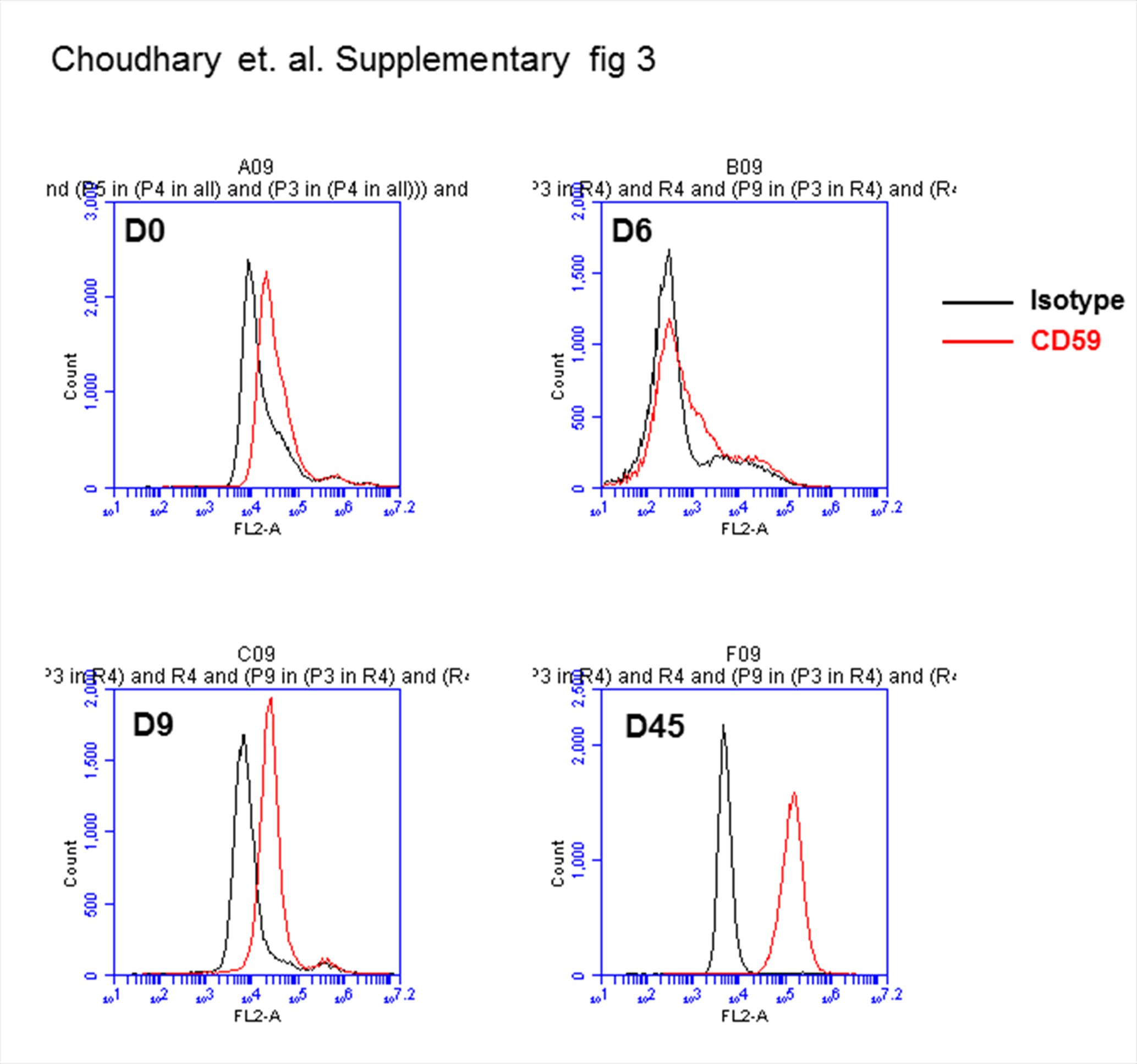

Supplement: Additional file 3: Figure S3. — Showing expression of CD59 measured by flow cytometry at days 0, 6, 9 and 45 of the differentiation time course. x axis represents the log-fluorescence intensity, y axis represents relative cell counts. (TIF 1704 kb) [file 13287_2016_380_MOESM3_ESM.tif]

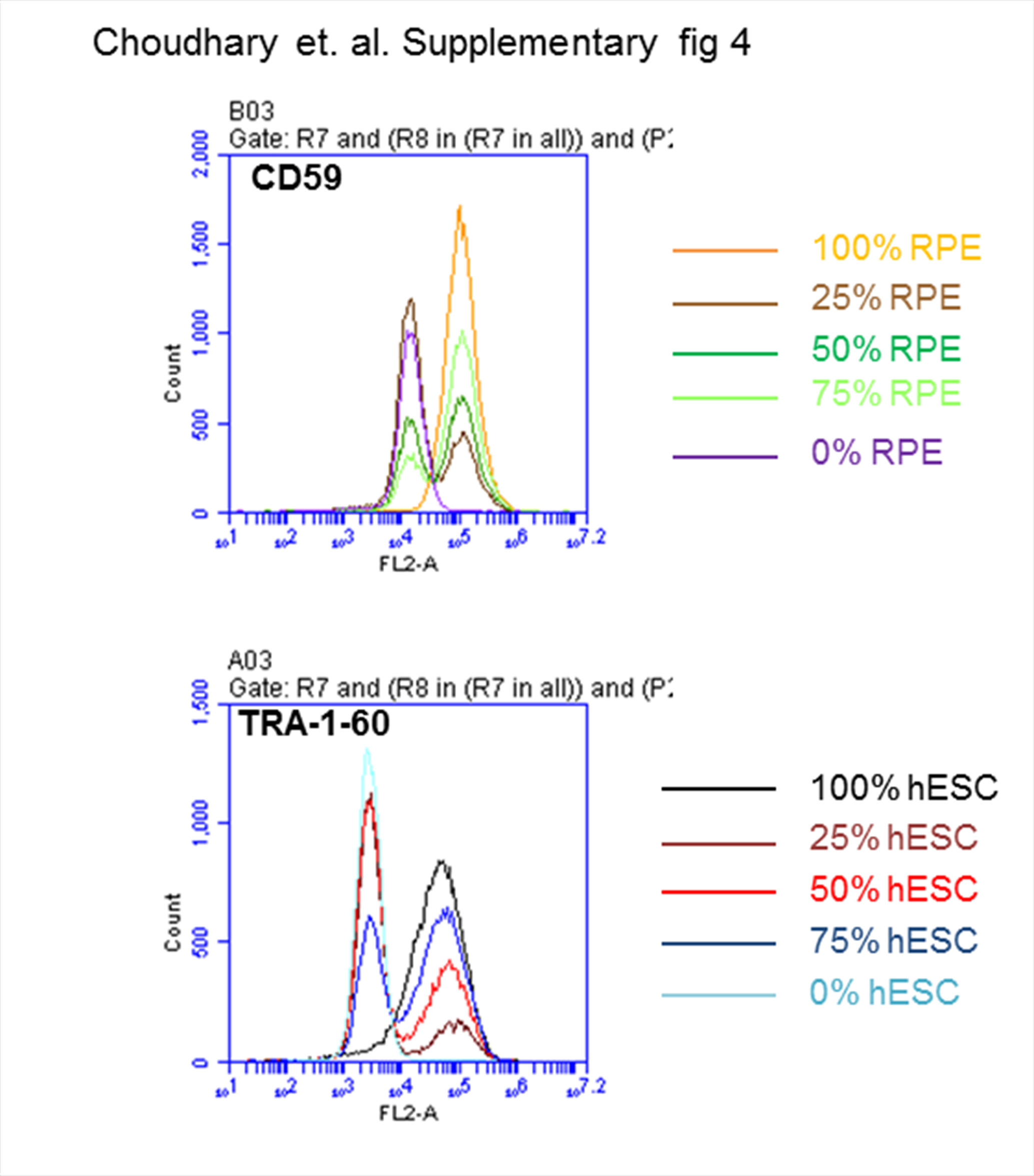

Supplement: Additional file 4: Figure S4. — Showing expression of CD59 (top) and TRA-1-60 (bottom) measured by flow cytometry in cell suspensions created by mixing together different hESC-derived RPE and hESCs. x axis represents the log-fluorescence intensity, y axis represents relative cell counts. (TIF 2661 kb) [file 13287_2016_380_MOESM4_ESM.tif]

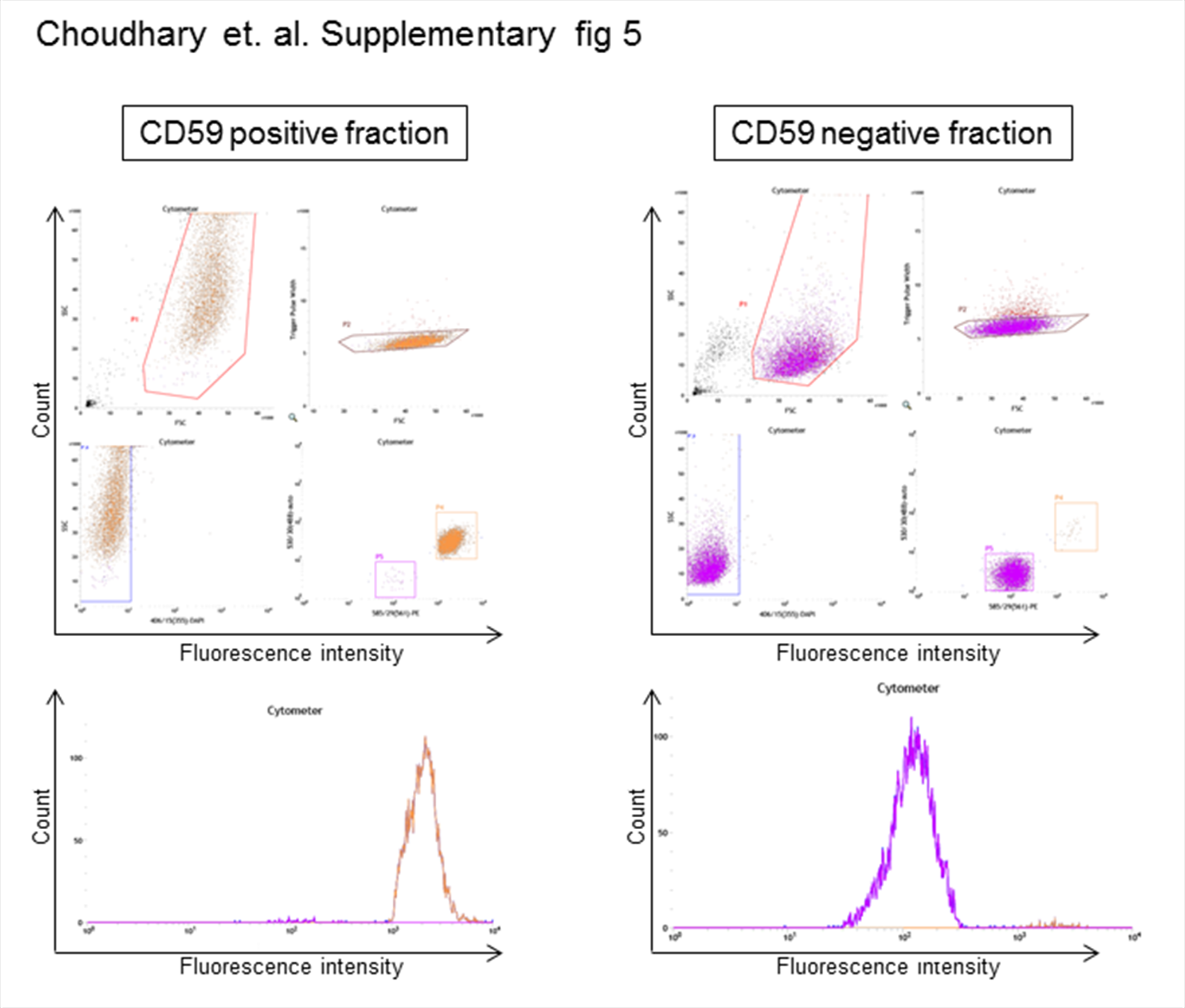

Supplement: Additional file 5: Figure S5. — Showing the CD59+ and CD59– samples sorted in Fig. 3 re-analysed by flow cytometry to show the purity of the individual fractions collected. The P4 gated events represent the CD59-positive fraction and the P5 gated events represent the CD59-negative fraction. (TIF 2445 kb) [file 13287_2016_380_MOESM5_ESM.tif]
